# The Role of Sodium Fluoride Mouthwash in Regulating FGF-2 and TGF-β Expression in Human Gingival Fibroblasts

**DOI:** 10.3390/biomedicines12081727

**Published:** 2024-08-01

**Authors:** Nobue Kato, Kumiko Nakai, Hideki Tanaka, Kyoko Fukuzawa, Minii Hayashi, Mikio Aoki, Takayuki Kawato

**Affiliations:** 1 Department of Oral Health Sciences, Nihon University School of Dentistry, Tokyo 1018310, Japan; deno21006@g.nihon-u.ac.jp (N.K.); tanaka.hideki@nihon-u.ac.jp (H.T.); kyoko.fukuzawa@gmail.com (K.F.); demi23023@g.nihon-u.ac.jp (M.H.); demi24001@g.nihon-u.ac.jp (M.A.); kawato.takayuki@nihon-u.ac.jp (T.K.); 2 Division of Functional Morphology, Dental Research Center, Nihon University School of Dentistry, Tokyo 1018310, Japan

**Keywords:** extraction sockets, FGF-2, mouthwash, sodium fluoride, TGF-β, wound healing

## Abstract

Sodium fluoride (NaF) is a fluoride application recommended by the World Health Organization for its efficacy and safety in preventing dental caries. Gingival fibroblasts that constitute the majority of connective tissue cells play a major role in wound healing via the expression of growth factors, including fibroblast growth factor-2 (FGF-2) and transforming growth factor-beta (TGF-β). This study examined the effect of NaF mouthwash on FGF-2 and TGF-β expression in human gingival fibroblasts (HGnFs). Fibroblasts were exposed to a medium with 225 ppmF NaF for 1 min, then switched to either 15 ppmF NaF for continuous stimulation or no NaF for transient stimulation. Continuous NaF stimulation significantly increased the gene and protein expression of FGF-2 and TGF-β in HGnFs compared to controls, suggesting NaF’s potential role in modulating periodontal tissue wound healing. Signaling pathway investigations showed the involvement of heterotrimeric GTP-binding proteins, calcium/calmodulin-dependent kinase II (CaMKII), and extracellular signal-regulated kinase (ERK) phosphorylation. Inhibiting CaMKII reduced NaF-induced FGF-2 and TGF-β expression, while ERK phosphorylation increased after NaF stimulation. These results highlight NaF mouthwash’s potential in promoting wound healing in extraction sockets, particularly during the mixed dentition period. Understanding NaF’s effects is clinically relevant due to the common use of fluoride products.

## 1. Introduction

The efficacy and safety of fluoride application (via toothpaste, mouthwash, dental application, etc.) for caries prevention have been well established by numerous domestic and international studies [1,2,3,4]. Fluoride, a naturally occurring mineral, plays a crucial role in the re-mineralization of enamel and the inhibition of demineralization, thereby significantly reducing the incidence of dental caries. Various methods of fluoride application, including toothpaste, mouthwash, and professional dental treatments, have been explored and endorsed globally for their effectiveness. Of these methods, mouthwash stands out as a fluoride application recommended by the World Health Organization because of its effectiveness, safety, and favorable cost–benefit ratio in preventing dental caries along with other health-economic aspects.

The adoption of fluoride mouthwash has been extensively studied, revealing its substantial impact on caries prevention. For instance, Torrell [5] reported that the daily use of a 0.05% sodium fluoride mouth rinse reduced the development of new caries by 50%, while rinsing every fortnight decreased caries incidence by approximately one-third. This study has become a classic model in the field of fluoride mouth rinsing. Subsequent studies have identified the use of a sodium fluoride mouthwash, starting from approximately 4 years of age, as one of the most important measures for early childhood caries prevention [6,7,8,9]. These findings underscore the significance of early and consistent fluoride exposure in oral-health maintenance and caries prevention in young children. During this period, primary teeth are replaced by permanent teeth. Therefore, the wound surface in gingival tissue can be exposed to fluoride after mouth rinsing. Permanent dentition also needs fluoride application to prevent caries. Therefore, the same situation is expected in adolescents with wounds caused by third-molar extraction or athletic injury. Specifically, the wound healing process after a tooth extraction is intricate and dynamic, with the aim of restoring the tissue to its original state. This process encompasses both epithelial regeneration and connective tissue formation, adhering to the general principles of wound healing observed in other tissues. The process is divided into several phases: inflammatory, proliferative, and maturation/remodeling. The proliferative phase, which begins approximately on the 4th day and can last up to 2 weeks post-injury, includes key activities, such as re-epithelialization, angiogenesis, granulation tissue formation, and collagen deposition [10]. The course of wound healing significantly influences the occurrence of subsequent complications [11], playing a crucial role in determining the overall outcome of the healing process. Thus, understanding the various stages of wound healing and the factors that can affect each stage is essential for effectively preventing and managing complications.

Gingiva is covered by stratified squamous epithelium with architectural characteristics unique to dental areas. Gingival fibroblasts constitute the majority of connective tissue cells under the epithelium and are believed to be responsible for the constant functional adaptation of the gingival connective tissue [12]. Therefore, gingival fibroblasts play a major role in wound healing, repair, and regeneration, particularly in the extraction fossa. These cells are pivotal in orchestrating the healing process, contributing to tissue remodeling and repair through various cellular mechanisms and signaling pathways [13,14]. Fibroblast growth factor-2 (FGF-2), which is important for gingival fibroblast proliferation, has recently been the focus of attention in periodontal tissue regeneration therapy. The use of drugs containing FGF-2 as an active ingredient has been confirmed to have regenerative effects on periodontal tissue [15,16]. The expression of FGF-2 and transforming growth factor-beta (TGF-β) produced by fibroblasts in wound healing promotes ECM synthesis and epithelial formation by acting on epithelial cells [17,18,19,20]. Thus, the function of gingival fibroblasts is essential in wound healing, and understanding and regulating their activity is crucial for optimizing healing outcomes.

Despite evidence indicating the role of low-concentration sodium fluoride (NaF) in the proliferation of human gingival cells, no studies have been conducted to elucidate the role and mechanism of action of NaF in wound healing [20]. This gap in knowledge highlights a critical area for further research, especially considering the widespread use of fluoride mouthwash in both pediatric and adult populations. Understanding the interaction between NaF and gingival fibroblasts during the wound healing process could provide valuable insights into the optimization of post-extraction care and improvement of overall oral-health outcomes.

Therefore, in this study, we aimed to investigate the effect of NaF mouthwash on extraction sockets after deciduous tooth decompensation through cell-based experiments. By focusing on the expression of FGF-2 and TGF-β in the presence of NaF, we sought to uncover potential benefits or adverse effects that could inform clinical practices and guidelines for fluoride use in dental care. This research is anticipated to bridge the existing knowledge gap and contribute to a more comprehensive understanding of fluoride’s role in oral wound healing.

## 2. Materials and Methods

### 2.1. Reagents

The reagents utilized in this study were procured from reputable sources. High-glucose (25 mM) Dulbecco’s modified Eagle’s medium (DMEM) and fetal bovine serum (FBS) were obtained from Gibco-BRL (Rockville, MD, USA), while fibroblast growth supplement (FGS) was sourced from ScienCell Research Laboratories (Carlsbad, CA, USA). Additionally, innovative antibiotic and antimycotic solutions (ZellShield^®^ is also active against mycoplasma) were acquired from Minerva Biolabs (Berlin, Germany). The specific kinase inhibitor KN-93 was obtained from Selleck Chemicals (Houston, TX, USA).

For protein analysis, the Proteome Profiler Human XL Cytokine Array Kit was purchased from R&D Systems (Minneapolis, MN, USA), and enzyme-linked immunosorbent assay (ELISA) kits for fibroblast growth factor (FGF), basic LAP (TGF-β), anti- extracellular signal-regulated kinase (ERK)1/2, and anti-Phospho-ERK1/2 were procured from R&D Systems (Minneapolis) and RayBiotech (Peachtree Corners, GA, USA), respectively.

### 2.2. Experimental Procedures

#### 2.2.1. Stimulation with NaF

Human gingival fibroblasts (HGnFs; Lot no. 25426; ScienCell Research Laboratories, Carlsbad, CA, USA) were seeded in a six-well plate at a density of 9 × 10^4^ cells/well. The cells were cultured in high-glucose DMEM supplemented with 2% FBS, 1% FGS, and 1% antibiotic and antimycotic solution (*v*/*v*) at 37 °C in 5% CO_2_ [21].

**Figure 1:**
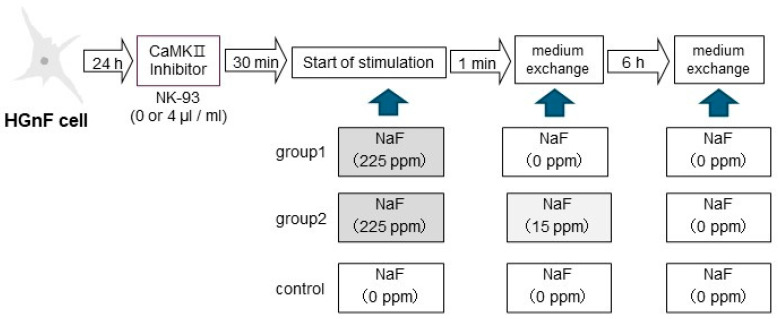
Based on the data from previous studies, the fibroblasts were exposed to a medium containing 225 ppmF NaF for 1 min, followed by replacement with a medium containing either a reduced NaF concentration (15 ppmF) for continuous stimulation (Group 2) or a medium without NaF for transient stimulation (Group 1).

A study that measured NaF concentration in plaque after a fluoride rinse showed that the fluoride concentration decreased with time, falling from 225 ppmF to approximately 15 ppmF at 1 min after rinsing and to the pre-rinse concentration after 6 h [22]. The stimulation concentration and stimulation time were determined based on previous research data (Figure 1).

Cell viability was measured by Cell Counting Kit-8 (Dojindo, Kumamoto, Japan) at 3, 6, and 24 h after stimulation to determine the cytotoxicity of NaF.

#### 2.2.2. Cytokine Array

Cell-free supernatants collected after 24 h from stimulated fibroblast cells were subjected to analysis using the Proteome Profiler Human XL Cytokine Array Kit following the manufacturer’s instructions. Cytokine levels were quantified from the array using the Image J Macro, Band/Peak Quantification Tool (US NIH, Bethesda, MD, USA).

#### 2.2.3. Real-Time Reverse Transcription (RT)-Polymerase Chain Reaction (PCR)

Total RNA was isolated from cells stimulated with/without NaF using the NucleoSpin^®^ RNA Kit (Takara Bio, Otsu, Japan) according to the manufacturer’s instructions. After measuring the concentration of total RNA using a NanoDrop 1000 spectrophotometer (Thermo Fisher Scientific, Waltham, MA, USA), cDNA was synthesized from 1 μg RNA using the PrimeScript™ RT Master Mix (Takara Bio). The resulting cDNA mixture was diluted 1:2 in sterile distilled water, and 2 µL of the diluted cDNA mixture was subjected to real-time RT-PCR with TB Green Premix Ex Taq™. The reaction mixture comprised 14 μL of TB Green Premix Ex Taq™ solution containing 8 μM sense and antisense primers, and the reactions involved 40 cycles at 95 °C for 5 s and 60 °C for 30 s on a CFX Connect Real-Time PCR system (Bio Rad, Hercules, CA, USA). To analyze gene expression, relative quantification was performed with respect to 18S rRNA expression using the CFX Manager™ Software1.1 [15,17].

PCR primers used in the experiments were as follows:

18S rRNA (F): 5′-CGGCTACCACATCCAAGGAA-3′, (R): 5′-CTGGAATTACCGCGGCT-3′

FGF-2 (F): 5′-CCCAGTTCGTTTCAGTGCCACC-3′, (R): 5′-CATTCAAAGGAGTGTGTGCA-3′

TGF-β (F): 5′-GCCCTGGACACCAACTATTG-3′, (R): 5′-GCACTTGCAGGAGCGCA-3′

#### 2.2.4. ELISA

FGF-2, TGF-β: Protein levels in culture supernatants and cell lysate with extraction buffer containing 0.5 mM phenylmethylsulfonyl fluoride, 0.05% Triton X-100, 0.5 mM ethylenediaminetetraacetic acid, and 25 mM Tris–HCl (pH 7.4) were quantified using ELISA kits following the manufacturer’s instructions. Specifically, ELISA kits from R&D Systems were employed for this purpose. To determine the concentration of each target protein, standard curves were generated using control peptides provided in the kits. These standard curves served as reference points against which the absorbance values of the samples were compared, enabling the accurate calculation of protein concentrations in both supernatants and lysates.

ERK1/2 phosphorylation: HGnF cells (20,000 cells per well) were seeded in 96-well plates and cultured overnight. The Ca^2+^/calmodulin-dependent protein kinase II (CaMKII) blocker KN93 was added 30 min prior to NaF stimulation at 225 ppmF. Next, a fixing solution was added at 5, 10, 15, and 30 min after stimulation to fix cells. Cells were reacted according to the kit manual and measured at 450 nm. The Anti-Phosph ERK1/2 value was divided by the Anti-ERK1/2 value to determine the degree of phosphorylation.

#### 2.2.5. Staining of Collagen and Non-Collagen Proteins

After washing with phosphate-buffered saline, cells underwent fixation by immersion in Kahle’s fixative, comprising ethanol, formaldehyde, and glacial acetic acid, for 10 min at 25 ± 1 °C. Subsequently, the fixed cells were immersed in the dye solution from the Sirius Red/Fast Green Collagen Staining Kit (Chondrex) and incubated for 30 min. Following the incubation period, the dye was eluted from samples for the semi-quantitative analysis of collagen and non-collagen proteins. OD values were measured at 540 nm (collagen) and 605 nm (non-collagen) utilizing a spectrophotometer. Then, the amount of collagen and non-collagenous protein was calculated using OD values, according to manufacturer guidelines [15].

### 2.3. Statistical Analysis

Data are presented as means ± standard deviations and were analyzed using GraphPad Prism (GraphPad Software 8.4.3, Boston, MA, USA). Statistical significance was determined using the *t*-test and one-way analysis of variance with Tukey’s multiple comparison test. Differences with *p*-values of <0.05 were considered statistically significant [21,23].

## 3. Results

### 3.1. Effect of NaF Stimulation on Cytokine Expression in HGnFs

**Figure 2:**
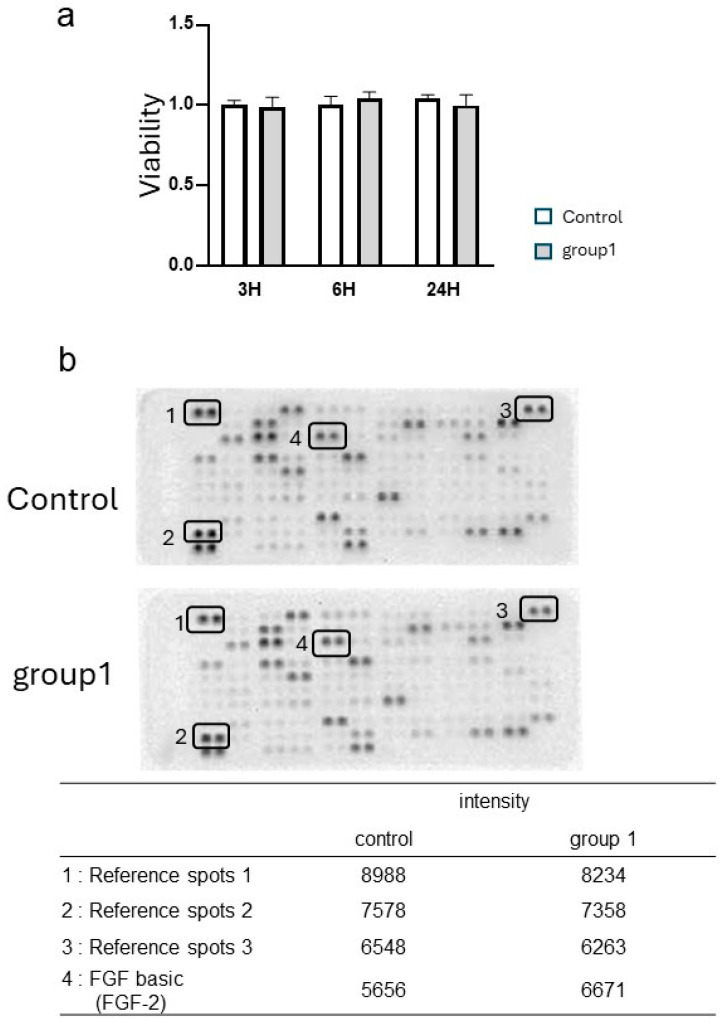
Impact of brief stimulation with sodium fluoride (NaF) on cell viability and cytokine expression in human gingival fibroblasts (HGnFs). HGnFs were exposed to 225 ppmF NaF stimulation for 1 min (Group 1). Subsequently, cell viability was quantified using the Cell Counting Kit-8 for cultured for 24 h and plotted as 1 for the control (**a**). Five wells per treatment. Each bar represents the mean ± standard deviation of three independent experiments. Cytokine expression levels in the culture supernatant were assessed at 24 h post-stimulation (**b**). Images were analyzed using Image J software and quantified using the Macro, Band/Peak Quantification Tool.

HGnFs were subjected to stimulation with 225 ppmF NaF for 1 min, and subsequent measurements of cell viability were conducted using the Cell Counting Kit-8 assay at 3, 6, and 24 h post-stimulation. Remarkably, no significant impact on cell viability was discernible (Figure 2a), thereby affirming the appropriateness of the NaF stimulation concentration. Following this validation, the expression of cytokines in the cell-culture supernatant at the 24 h mark post-stimulation with 225 ppmF NaF was scrutinized using The Proteome Profiler Human XL Cytokine Array Kit. Notably, spots indicative of FGF-2 expression exhibited enhancement upon stimulation with 225 ppmF NaF compared to the control (Figure 2b); this finding was duly substantiated by quantification utilizing Image J software https://www-p.sci.ocha.ac.jp/ohganelab/2022/08/08/imagej (accessed on 26 July 2024).

### 3.2. Effect of NaF Stimulation on FGF-2 and TGF-β Expression in HGnFs

**Figure 3:**
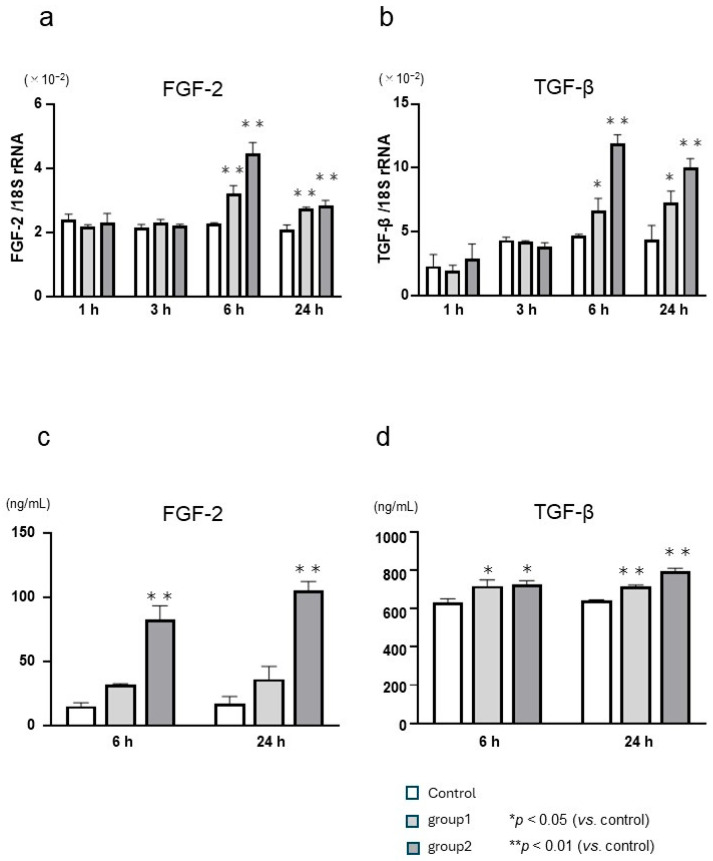
Influence of short-term and continuous sodium fluoride (NaF) stimulation on the mRNA and protein expression of fibroblast growth factor-2 (FGF-2) and transforming growth factor beta (TGF-β) in human gingival fibroblasts (HGnFs). HGnFs were stimulated with 225 ppmF NaF for 1 min (Group 1) followed by culture for 24 h or 225 ppmF NaF for 1 min followed by stimulation with 15 ppm NaF for culture for 24 h (Group 2). mRNA expression of FGF-2 and TGF-β was quantified using real-time polymerase chain reaction (**a**,**b**), while protein expression was quantified using enzyme-linked immunosorbent assay (**c**,**d**). Five wells were used per treatment. Each bar indicates the mean ± standard deviation of three independent experiments.

The mRNA and protein levels of FGF-2 and TGF-β in cells stimulated with 225 ppmF NaF were examined at 1, 3, 6, and 24 h post-stimulation using real-time PCR (Figure 3a,b) and ELISA (Figure 3c,d), respectively. After 6 and 24 h of culture, *FGF-2* gene expression increased in cells subjected to short-term (Group 1) and continuous (Group 2) stimulation with NaF compared with unstimulated control cells, with a significant difference being observed especially in Group 2. *TGF-β* gene expression also showed a significant difference compared to controls in both Groups 1 and 2. FGF-2 and TGF-β protein levels were significantly higher in both Groups 1 and 2 compared to controls.

### 3.3. Effect of NaF Stimulation on Collagen and Non-Collagen Expression in HGnFs

**Figure 4:**
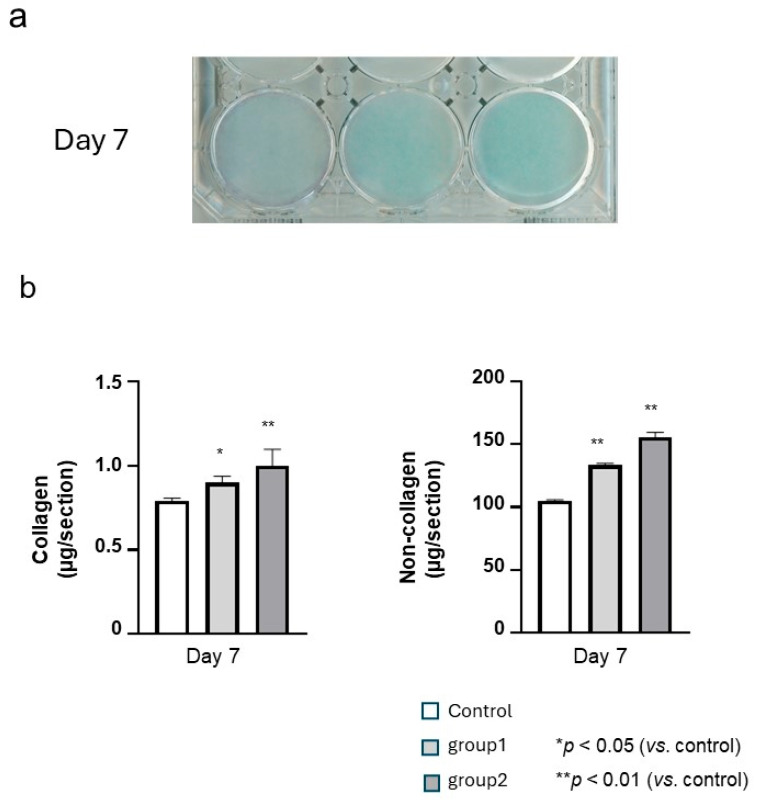
Influence of short-term and continuous sodium fluoride (NaF) stimulation on the mRNA and protein expression of collagen and non-collagen proteins in human gingival fibroblasts (HGnFs). HGnFs were exposed to NaF stimulation for 7 days. Collagenous (red) and non-collagenous (green) proteins were examined by Sirius Red and Fast Green staining, respectively, on day 7 of the culture (**a**), and quantitative analysis (**b**) was conducted using the dye extraction buffer. Five wells were used per treatment. Each bar indicates the mean ± standard deviation of three independent experiments.

Collagenous and non-collagenous proteins in the cultured cells were stained with Sirius Red and Fast Green on day 7 of culture, respectively. The deposition of non-collagenous (green) proteins was observed in both groups stimulated with NaF and control cells. In contrast, it was difficult to confirm collagenous (red) deposition in microscopic images (Figure 4a). However, quantitative analysis revealed that the amounts of collagenous and non-collagenous proteins were significantly higher after NaF stimulation than in the unstimulated control (Figure 4b).

### 3.4. Effect of NaF and/or KN93 on the Phosphorylation of ERK1/2

**Figure 5:**
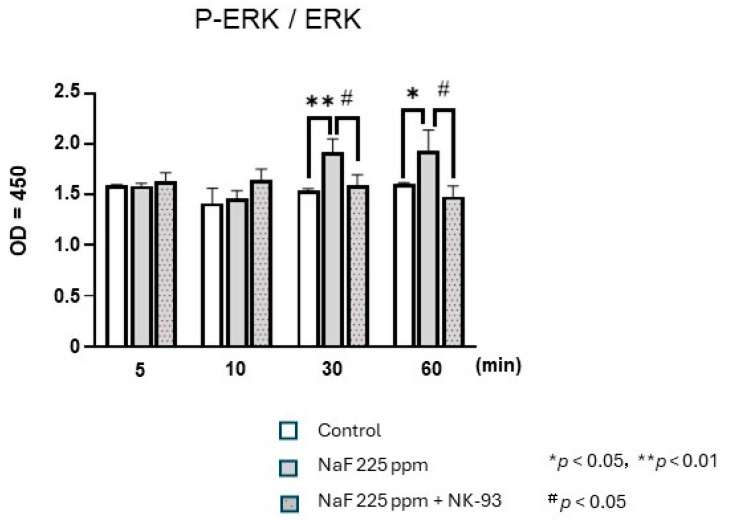
Sodium fluoride stimulation of human gingival fibroblasts in the presence or absence of 5 μM KN93 (inhibitor of Ca^2+^/calmodulin-dependent protein kinase II) was performed in six-well plates for 1 min, and phosphorylation of ERK1/2 after 5, 10, 30, and 60 min was examined using enzyme-linked immunosorbent assay. Each bar indicates the mean ± standard deviation of four independent experiments. * *p* < 0.05, ** *p* < 0.01, # *p* < 0.05. ERK, extracellular signal-regulated kinase.

ERK1/2 phosphorylation exhibited a notable increase at both 30 and 60 min following NaF treatment. Intriguingly, the administration of NK93, which is known to be a CaMKII inhibitor, resulted in the complete blockade of the NaF-induced stimulatory effects on ERK1/2 phosphorylation at the 30 and 60 min time points (Figure 5).

### 3.5. Effect of NK93 on the Expression of FGF-2 and TGF-β Induced by NaF

**Figure 6:**
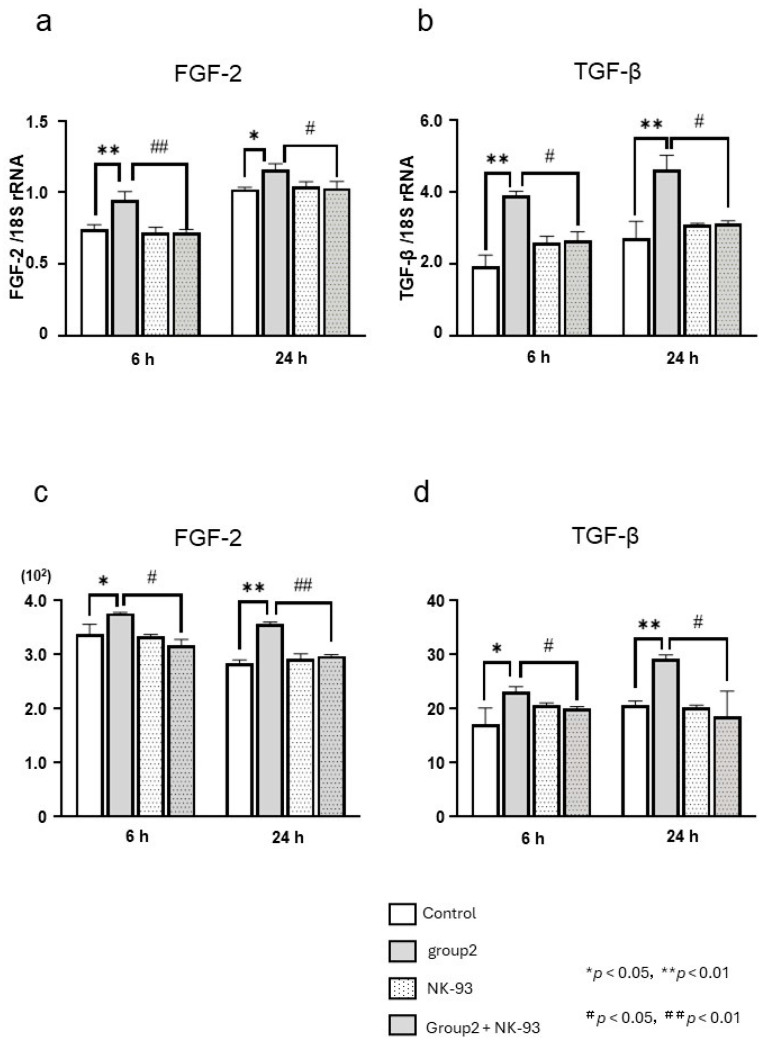
Effect of KN93 on sodium fluoride (NaF)-induced fibroblast growth factor-2 (FGF-2) and transforming growth factor beta (TGF-β) expression. Human gingival fibroblasts were cultured in six-well plates with or without 5 µM KN93 (inhibitor of Ca^2+^/calmodulin-dependent protein kinase II (CaMKII) in the presence or absence of NaF for 24 h, and FGF-2 and TGF-β mRNA levels in the cells were determined by real-time polymerase chain reaction (**a**,**b**). The FGF-2 and TGF-β protein levels in the cells were measured by enzyme-linked immunosorbent assay (**c**,**d**). Six wells were used per treatment. Each bar indicates the mean ± standard deviation of three independent experiments. * *p* < 0.05, ** *p* < 0.01, # *p* < 0.05. ## *p* < 0.01.

NK93 effectively suppressed the stimulatory effect induced by NaF on the expression of FGF-2 and TGF-β mRNA (Figure 6a,b). Moreover, NK93 exhibited a complete inhibition of the stimulatory effect of NaF on the expression of FGF-2 and TGF-β proteins (Figure 6c,d).

## 4. Discussion

In this study, we investigated the effects of NaF, a component of fluoride mouthwash, on the expression of FGF-2 and TGF-β in fibroblasts. Short-term stimulation with 225 ppmF NaF induced the upregulation of FGF-2 and TGF-β; this upregulation was more dominant on the continuous stimulation of NaF at a reduced concentration of 15 ppmF. This suggests that FGF-2 may enhance fibroblast growth, while TGF-β may modulate the regulatory function of periodontal tissue during the wound healing process when using NaF mouthwash.

Numerous epidemiological, clinical, and cytogenetic studies have been conducted on fluoride toxicity [24,25]. The cytotoxicity of NaF in agents used in oral care is significantly correlated with time and concentration, with previous studies indicating that the concentration threshold for fluoride is 400 ppm and that it is not toxic to fibroblasts even after 30 min of exposure [25]. In the present study, no effect of NaF stimulation on cell viability was observed. This is likely attributed to the low concentration of NaF used in this study (225 ppmF) and the short duration of action (1 min). Wang et al. [26] also reported that low concentrations of NaF may promote soft tissue healing by inducing fibronectin (FN) and laminin, while Bhawal et al. [27] noted that the increased expression of Runx2 and osteocalcin may promote hard tissue regeneration. The authors also reported that the increased expression of Runx2 and osteocalcin may promote hard tissue regeneration. These reports also indicate that low concentrations of NaF are involved in the healing and regeneration of various cells, and similar effects may be observed in fibroblasts.

Fibroblasts, a key cellular component in the remodeling of periodontal tissue, play an essential role in the wound healing process. They synthesize and organize various extracellular matrix (ECM) proteins, including collagen, non-collagenous proteins, elastin, and proteoglycans. The ECM is vital for providing the structural support and biochemical cues necessary for tissue repair and regeneration, and ECM production is deeply associated with growth factors, including FGF. The FGF family comprises 23 members, of which FGF-2, FGF-7, and FGF-10 are crucial for cutaneous wound healing [28,29,30,31]. These growth factors are produced by keratinocytes, fibroblasts, and endothelial cells. In particular, FGF-2, also known as basic FGF, is upregulated during the acute phase of wound healing and contributes to granulation tissue formation, re-epithelialization, and tissue remodeling [32,33,34]. In vitro studies have demonstrated its role in regulating the synthesis and deposition of various ECM components, promoting keratinocyte motility, and stimulating fibroblast migration and collagenase production [35].

TGF-β family comprises a diverse group of cytokines, including TGF-β1, TGF-β2, and TGF-β3, as well as bone morphogenic proteins and activins. Among these, TGF-β1 stands out as the predominant isoform involved in cutaneous wound healing. This particular growth factor is produced by macrophages, fibroblasts, keratinocytes, and platelets, indicating its widespread influence in the wound healing process [36,37,38,39]. TGF-β1 plays a multifaceted role in orchestrating the complex series of events that occur during wound healing. One of its primary functions is in modulating the inflammatory response, which is essential for preventing infection and clearing debris from the wound site. During the initial phase of wound healing, TGF-β1 attracts immune cells, such as macrophages, to the wound site, thereby setting the stage for subsequent healing processes [40]. Re-epithelialization, a process where new epithelial cells cover the wound, is also significantly influenced by TGF-β1. It stimulates keratinocyte migration and proliferation, accelerating the restoration of the skin’s protective barrier. Moreover, TGF-β1 is heavily involved in connective tissue regeneration, particularly in the formation of granulation tissue [41], which is initiated by TGF-β1 through the upregulation of genes related to the ECM. This leads to the increased synthesis and deposition of ECM components, such as collagen and FN, which provide structural integrity to the healing tissue. Additionally, TGF-β1 facilitates wound contraction by stimulating fibroblasts to contract the collagen matrix, thereby reducing the wound size and expediting closure [42,43,44,45]. In this study, we observed the elevated production of ECM and TGF in NaF-stimulated cells. The aforementioned findings and our present results indicate potential therapeutic approaches using NaF mouthwash that aim to promote wound healing and improve clinical outcomes.

The early activation of FGF-2 and TGF-β during periodontal tissue wound healing, particularly in regenerating gingival connective tissue, promotes FN production and cell migration and proliferation, thereby significantly influencing ECM synthesis, maturation, and granulation tissue organization [19]. Our study revealed an increase in both gene and protein expressions of FGF-2 and TGF-β after 6 and 24 h of NaF stimulation compared to those of the control group. This suggests that NaF-induced TGF-β1 and FGF-2 expression accelerates the wound healing process in its early stage.

ECM contains collagenous and non-collagenous proteins. In the present study, NaF stimulation increased collagen and non-collagen staining compared to that in controls. Collagen provides structural support in the extracellular space of connective tissues; furthermore, collagen fibers are reported to not accumulate in the absence of FN, which is a non-collagenous protein [46,47,48]. These findings suggest that the FN matrix is an important scaffold for collagen assembly and that both collagen and non-collagenous proteins play important roles in wound healing. TGF-β has been widely studied as a fibrotic cytokine. It induces the production of ECM proteins, such as collagen and FN, in myofibroblasts and adipocytes [49,50,51,52]. Thus, it is possible that the NaF-stimulated increase in TGF-β increased the production of collagen. Moreover, AS mouthwashes containing 225 ppmF NaF are commonly used daily in Japan, and the accelerated healing process could potentially be maintained.

Within hours of an injury, epithelial remodeling is initiated, with the release of TGF-α, FGF, and other substances promoting epithelial cell migration and proliferation [53]. During tissue formation, keratinocytes migrate from the wound margin to the wound center in the regenerating granulation tissue, driven by growth factors and cytokines released from the injury site, resulting in re-epithelialization. Both FGF-2 and TGF-β, increased by NaF stimulation, may contribute to epithelial–mesenchymal transition and promote re-epithelialization [20]. NaF is a potent, rapid, and reversible activator of regulatory heterotrimeric GTP-binding proteins, which are crucial for transmitting signals from cell surface receptors to internal cellular pathways, in virtually all in vitro systems. These GTP-binding proteins play key roles in various cellular processes, including cell growth, differentiation, and metabolism [54].

**Figure 7:**
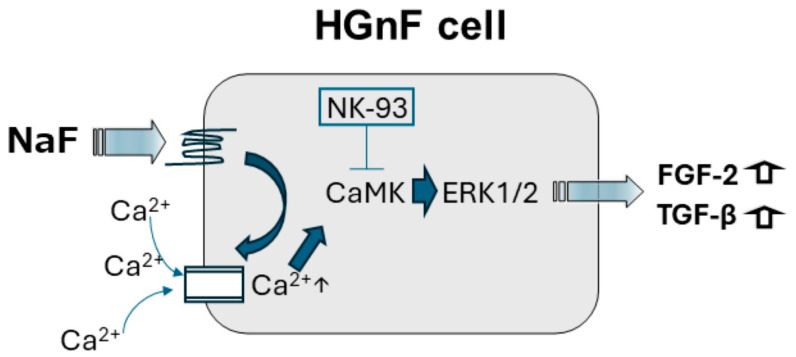
Based on the findings of previous studies and the present study, NaF stimulation of HGnF cells activates Gs under the G protein-coupled (7-transmembrane) receptor to promote Ca^2+^ influx into cells. Intracellular Ca^2+^ influx activates CaMKII, increasing downstream ERK phosphorylation and leading to increased FGF-2 and TGF-β expressions, both of which are suppressed by CaMKII inhibitor (NK-93).

G proteins on the plasma membrane of cells are classified into four families (Gs, Gi, Gq, and G12). NaF activates Gs signaling on fibroblasts, promoting adenylate cyclase activity and the opening of calcium ion channels, leading to Ca^2+^ influx and subsequent CaMKII activation [55]. Previous studies have demonstrated NaF-induced Gs activation and intracellular Ca^2+^ mobilization in rat glioma cells. Our study revealed phosphorylation of ERK at 30 and 60 min after NaF stimulation, indicating CaMKII involvement [54,56]. Moreover, the inhibition of CaMKII with KN93 attenuated NaF-induced ERK phosphorylation in our study, in consistency with the findings of Bogatcheva et al. [57]. Furthermore, our results show that the inhibition of CaMKII by NK93 suppressed the gene and protein expressions of FGF-2 and TGF-β. Ikushima et al. and Zhao et al. have already demonstrated that the expression of FGF-2 and TGF-β increased via the phosphorylation of ERK [58,59]. Therefore, the inhibition of CaMKII may attenuate the phosphorylation of ERK, suppressing the expressions of FGF-2 and TGF-β. These findings, coupled with those of our study, suggest that NaF stimulation of HGnFs increases FGF-2 and TGF-β expressions by activating Gs-coupled receptors, leading to CaMKII activation and subsequent ERK phosphorylation downstream of the receptor (Figure 7). This may contribute to fibroblast activation and ECM protein remodeling.

Previous findings regarding the functions of FGF-2 and TGF-β and our present results highlight the multifaceted role of NaF in the wound healing process, particularly in the context of dental-tissue regeneration. The dual effect of NaF on the expression of FGF-2 and TGF-β underscores the complex interplay between different signaling pathways and growth factors in promoting tissue repair and regeneration. The upregulation of these key growth factors suggests that NaF enhances fibroblast proliferation and modulates the regulatory environment of the periodontal tissue, potentially leading to more effective and accelerated wound healing. Our findings emphasize the potential clinical implications of using NaF mouthwash in routine dental care. The ability of NaF to enhance wound healing processes through the activation of key signaling pathways could be particularly beneficial in managing post-extraction care and other periodontal treatments. Given the widespread use of NaF mouthwash in Japan and other parts of the world, these results provide a scientific basis for its continued use and potential enhancement of dental health practices.

While this study provides valuable insight into the cellular mechanisms underlying wound healing, a few limitations must be acknowledged. First, in vitro cell-culture models may not fully reproduce the complex environment of the in vivo tissue environment. In addition, the cell lines used herein may not fully represent the heterogeneity of cell populations found in vivo. Therefore, cell behavior and interactions may be oversimplified. Second, the influence of systemic factors, such as immune response and hormonal influences, is not considered in studies using isolated cells. These factors play an important role in wound healing and tissue regeneration. Therefore, while the findings herein advance our understanding of cellular processes, further research using animal models and clinical samples is essential to validate these results and extend them to a more comprehensive physiological framework.

Future studies should aim to further elucidate the precise molecular mechanisms underlying NaF’s effects on wound healing. Moreover, epithelial cells and bone marrow mesenchymal stem cells are also associated with wound healing [27]. Additionally, in vivo studies could provide more comprehensive insights into how NaF influences tissue regeneration in a complex biological environment. Understanding these mechanisms in greater detail could pave the way for the development of new therapeutic strategies that leverage NaF’s beneficial properties for improved dental and periodontal health.

## Data Availability

Data contained within the article.
